# Edward Calvin Kendall: A Pioneer in Biochemistry and Endocrinology

**DOI:** 10.7759/cureus.69420

**Published:** 2024-09-14

**Authors:** Nadjib Kaouache, Nassim Nouri

**Affiliations:** 1 Internal Medicine, University Hospital of Béjaïa, Béjaïa, DZA; 2 Endocrinology and Diabetes, Faculty of Medicine of Constantine, Salah Boubnider University Constantine 3, Constantine, DZA

**Keywords:** cortisone discovery, edward calvin kendall, endocrinology, nobel prize 1950, thyroxine isolation

## Abstract

Edward Calvin Kendall was a distinguished American biochemist known for pioneering research on adrenal cortex hormones, notably cortisone. This review article examines Kendall's life, career, and key scientific contributions, including his groundbreaking work on thyroid hormones and corticosteroids. The discussion emphasizes Kendall's role in advancing biochemical research and his lasting influence on physiology and medicine. By analyzing primary sources and historical accounts, this article offers a thorough overview of Kendall's impact on biochemistry and endocrinology.

## Introduction and background

Edward Calvin Kendall was an influential biochemist whose research had profound implications for endocrinology and therapeutic practices in the 20th century. Born in South Norwalk, Connecticut, Kendall's scientific journey began at Columbia University, where he earned his PhD in 1910 [1]. His career was marked by pivotal discoveries that reshaped the understanding and treatment of hormonal disorders, particularly through his isolation of L-thyroxine (levothyroxine) and cortisone [2,3]. This article aims to provide a detailed account of Kendall's life, scientific achievements, and lasting impact on medical science.

## Review

Early life and education

Edward Calvin Kendall was born on March 8, 1886, in South Norwalk, Connecticut. His formative years were spent in a supportive educational environment that nurtured his early interest in science. Attending local schools, Kendall demonstrated a keen aptitude for academics, which paved the way for his higher education pursuits. In 1904, he entered Columbia University, one of the leading institutions of the time. At Columbia, Kendall earned his Bachelor of Science degree in 1908, followed by a Master of Science degree in 1909. His academic journey culminated in 1910 with a PhD, with his thesis focusing on the kinetics of pancreatic amylase, an enzyme crucial for starch digestion [3,4]. Kendall’s doctoral research on enzyme kinetics laid the groundwork for his future contributions to biochemistry. His early work not only provided insights into enzyme behavior but also demonstrated his ability to tackle complex biochemical problems. This foundational work foreshadowed his later achievements in hormone isolation and endocrine research, setting the stage for his illustrious career [2].

Early career and contributions

Kendall’s professional career began at Parke, Davis & Co. in Detroit, Michigan, where he was assigned the challenging task of isolating thyroid hormones. This role was a significant test of his scientific skills and perseverance. Despite the complexities involved in isolating and characterizing thyroid hormones, Kendall’s dedication led to notable progress in the field. His early success at Parke, Davis & Co. established him as a promising researcher in biochemistry [5]. In 1911, Kendall transitioned to St. Luke's Hospital in New York City, where he continued his research on thyroid extracts. His work at St. Luke's allowed him to refine his techniques and further his understanding of thyroid function. However, it was his move to the Mayo Foundation in Rochester, Minnesota, in 1914 that marked the beginning of his most influential phase [6].

At the Mayo Foundation, Kendall embarked on a research journey that defined his career. His time at Mayo was marked by intense scientific exploration and collaboration, leading to one of his most significant achievements: the isolation of thyroxine from the thyroid gland. By December 1914, Kendall had succeeded in isolating thyroxine in crystalline form, a landmark accomplishment that represented the first successful isolation of a pure hormone [7].

Isolation and study of thyroxine

The isolation of thyroxine was a groundbreaking achievement in endocrinology. Kendall’s method involved the alkaline hydrolysis of thyroid tissue, followed by an iodine assay. This approach allowed him to trace and purify the hormone through various stages, ultimately leading to the successful isolation of crystalline thyroxine. Initially, Kendall referred to the substance as "iodine-A," but he later adopted the accepted nomenclature for clarity and consistency [8]. The successful isolation of thyroxine had profound implications for the study of thyroid function. It provided researchers with a pure substance for more accurate and detailed investigations into thyroid physiology. Furthermore, Kendall’s work highlighted the crucial role of iodine in thyroid health, contributing significantly to the understanding of iodine deficiency disorders and their impact on global health [9].

Research on adrenal corticosteroids

The 1930s marked a new phase in Kendall’s research, as he shifted his focus to the adrenal cortex. Collaborating with H. L. Mason and other researchers, Kendall’s team embarked on a project to isolate and characterize corticosteroids from adrenal extracts. This work was both complex and pioneering, involving the extraction and analysis of several biologically active compounds [2]. By 1935, Kendall’s team had identified six distinct corticosteroids, labeled alphabetically from A to F. Among these, Compound E emerged as the most significant. Compound E was later renamed cortisone and became central to Kendall’s research and clinical applications. The team’s efforts to elucidate cortisone’s structure and develop methods for its synthesis were crucial in advancing the understanding of adrenal gland function and its physiological effects [10,11].

Advocacy and clinical impact

The late 1940s saw a breakthrough in Kendall’s research, thanks to his collaboration with Philip Hench, a rheumatologist at the Mayo Clinic. Together, they discovered the profound effects of cortisone on patients with rheumatoid arthritis. This discovery was transformative, offering a new and effective treatment for a condition that had previously been difficult to manage [1]. The clinical application of cortisone represented a major advancement in the treatment of inflammatory diseases. It not only provided relief to patients suffering from rheumatoid arthritis but also paved the way for the development of corticosteroid-based therapies for a wide range of inflammatory and autoimmune disorders. The successful synthesis and application of cortisone marked a significant milestone in pharmacology and therapeutics, revolutionizing medical practice [12,13].

Legacy and impact

**Figure 1:**
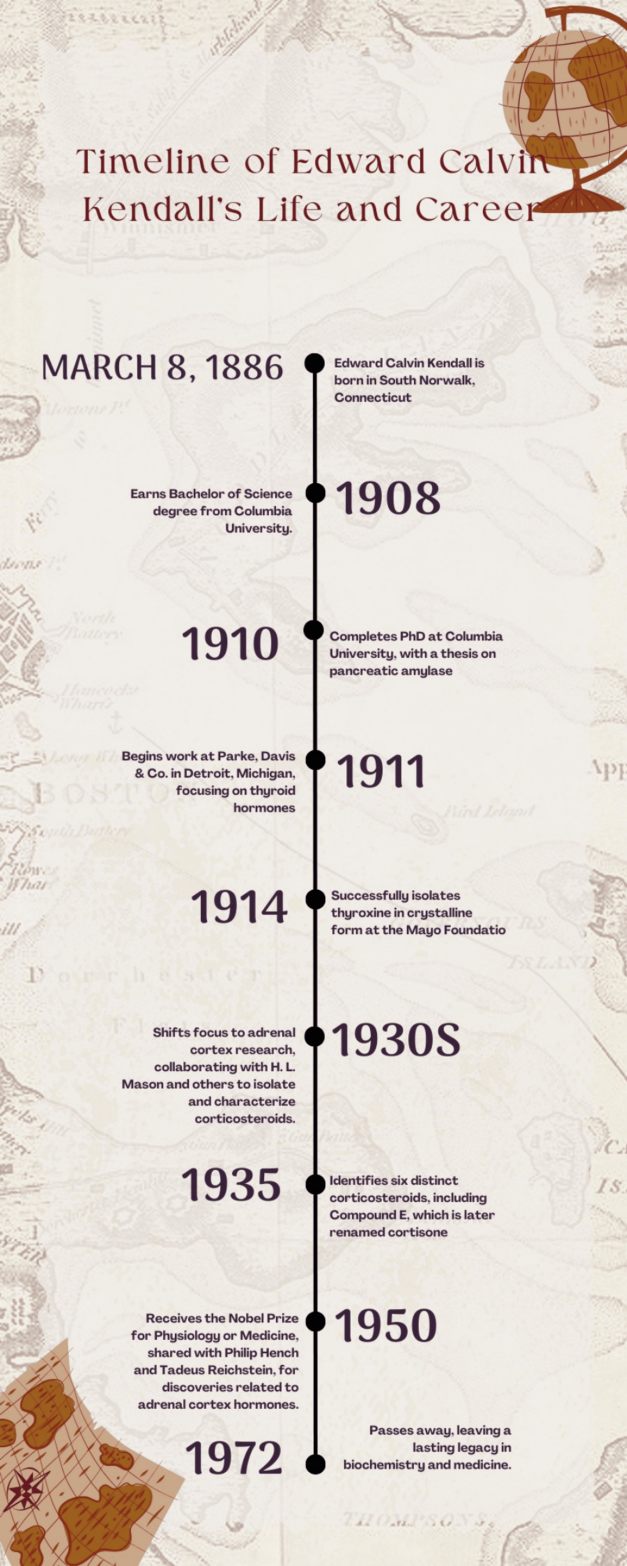
Timeline of Edward Calvin Kendall's Life and Career Image credit: Nadjib Kaouache

Edward Calvin Kendall’s contributions to biochemistry and medicine were globally recognized when he, along with Philip Hench and Tadeus Reichstein, received the Nobel Prize for Physiology or Medicine in 1950. The Nobel Prize was awarded in recognition of their "discoveries relating to the hormones of the adrenal cortex, their structure, and biological effects" [14]. Kendall’s pioneering work laid the foundation for modern endocrine therapy and biochemical research, influencing generations of scientists and clinicians. Kendall’s meticulous approach to hormone isolation and characterization set new standards in biochemical research. His work not only advanced the scientific understanding of hormonal functions but also had practical implications for the treatment of endocrine disorders. Moreover, his collaboration with clinicians like Hench exemplified the impact of translational research, demonstrating how scientific discoveries can lead to significant advancements in medical practice [15]. After retiring from the Mayo Clinic in 1951, Kendall continued his research as a visiting professor at Princeton University. He remained active in scientific discussions and writing, contributing to the field until he died in 1972. Kendall’s legacy is marked by his groundbreaking discoveries and the lasting impact of his work on both science and medicine [2]. His career serves as a testament to the importance of perseverance and innovation in advancing scientific knowledge and improving human health. To provide a clearer overview of Edward Calvin Kendall's significant contributions, a timeline summarizing the key milestones in his life and career is presented below (Figure 1).

## Conclusions

In conclusion, Edward Calvin Kendall's career epitomizes the profound impact of scientific innovation and dedication. His pioneering work in hormone isolation and characterization revolutionized biochemistry and medicine. Kendall's contributions, from thyroxine to cortisone, expanded therapeutic options and improved patient outcomes. His meticulous approach established a new standard in biochemical research. Today, Kendall’s legacy endures through ongoing advancements in endocrinology and pharmacology, reflecting the lasting significance of his discoveries.
